# Treatment of Neuralgia of the Neck of the Uterus by Incision of This Organ, by M. Malgaigne

**Published:** 1849-05

**Authors:** 


*Treatment of neuralgia of the neck of the uterus by incision of this organ, by M. Malgaigne.*—The author performs this incision by sliding a pair of scissors upon the index finger of the left hand until the neck of the uterus is reached, when one of the blades is introduced internal, the other external to the seat of the pain, and with a single stroke he divides the anterior lip at the point indicated. M. Malgaigne is said to have repeatedly performed this operation, and that twice it was followed by hemorrhage. In both these instances he had practiced the horizontal incision by means of the curved scissors. He has given up this procedure for the vertical incision which never causes the loss of more than a few drops of blood.

